# Glucagon-Like Peptide-1 Receptor Agonist Liraglutide Ameliorates the Development of Periodontitis

**DOI:** 10.1155/2020/8843310

**Published:** 2020-11-19

**Authors:** Noritaka Sawada, Kei Adachi, Nobuhisa Nakamura, Megumi Miyabe, Mizuho Ito, Shuichiro Kobayashi, Shin-ichi Miyajima, Yuki Suzuki, Takeshi Kikuchi, Makoto Mizutani, Taku Toriumi, Masaki Honda, Akio Mitani, Tatsuaki Matsubara, Keiko Naruse

**Affiliations:** ^1^Department of Periodontology, School of Dentistry, Aichi Gakuin University, Nagoya, Japan; ^2^Department of Internal Medicine, School of Dentistry, Aichi Gakuin University, Nagoya, Japan; ^3^Department of Oral Anatomy, School of Dentistry, Aichi Gakuin University, Nagoya, Japan

## Abstract

Periodontitis is one of the diabetic complications due to its high morbidity and severity in patients with diabetes. The prevention of periodontitis is especially important in diabetic patients because the relationship between diabetes and periodontitis is bidirectional. Here, we evaluated the impacts of glucagon-like peptide-1 (GLP-1) receptor agonist liraglutide on the amelioration of periodontitis. Five-wk-old Male Sprague–Dawley (SD) rats (*n* = 30) were divided into 3 groups: normal, periodontitis, and periodontitis with liraglutide treatment groups. Periodontitis was induced by ligature around the maxillary second molar in SD rats. Half of the rats were administered liraglutide for 2 weeks. Periodontitis was evaluated by histological staining, gene expressions of inflammatory cytokines in gingiva, and microcomputed tomography. Periodontitis increased inflammatory cell infiltration, macrophage accumulation, and gene expressions of tumor necrosis factor-*α* and inducible nitric oxide synthase in the gingiva, all of which were ameliorated by liraglutide. Liraglutide decreased M1 macrophages but did not affect M2 macrophages in periodontitis. Moreover, ligature-induced alveolar bone resorption was ameliorated by liraglutide. Liraglutide treatment also reduced osteoclasts on the alveolar bone surface. These results highlight the beyond glucose-lowering effects of liraglutide on the treatment of periodontitis.

## 1. Introduction

Periodontitis is a chronic multifactorial inflammatory disease that progresses and destroys periodontal tissue, including alveolar bone, due to periodontal pathogens [1]. The resorption of alveolar bone leads to tooth loss.

Periodontitis is considered one of the diabetic complications by the high morbidity and the severity in diabetes compared with the nondiabetic subjects [2–5]. Furthermore, the relation between diabetes and periodontitis is bidirectional. Meta-analyses demonstrated that the treatment of periodontitis reduced HbA1c in type 2 diabetes [6, 7]. An 11-year prospective observational study in Pima Indians reported that the more severe the periodontitis, the higher the mortality rate, especially from ischemic heart disease and diabetic nephropathy [8].

We previously demonstrated the beneficial effects of glucose-dependent insulinotropic polypeptide (GIP) on periodontitis via its anti-inflammatory effects using ligature-induced experimental periodontitis model mice [9]. GIP and glucagon-like peptide-1 (GLP-1) are incretin hormones which are secreted from the intestine. The primary role of incretin hormones is the stimulation of insulin secretion from pancreatic *β*-cells. However, since the receptors of incretin hormones are expressed in the whole body, extrapancreatic actions of incretin hormones are considered to play an important role [10]. In fact, the American Diabetes Association (ADA)/European Association for the Study of Diabetes (EASD) consensus report recommended GLP-1 receptor agonists for patients with cardiovascular disease because of their cardioprotective results from clinical megastudies [11]. Furthermore, GLP-1-based therapies were reported to have anti-inflammatory effects in several organs, including the heart, brain, kidney, liver, pancreas, skin, and testis [12]. *In vitro* study revealed the effects of GLP-1 on macrophage polarization [13]. GLP-1 also affects bone construction [14]. GLP-1-receptor-deficient mice displayed cortical osteopenia and bone fragility due to increased bone resorption with increased osteoclast number and bone resorption activity [15].

The anti-inflammatory and bone construction effects of GLP-1 make us expect the beneficial effects of GLP-1 on the development and progression of periodontitis. Since the extrapancreatic effects of GLP-1 do not depend on glycemic control, we hypothesized that GLP-1 may improve periodontitis with or without diabetes. In this study, we investigated the effects of the GLP-1 receptor agonist, liraglutide, on the development of periodontitis.

## 2. Materials and Methods

### 2.1. Animals

Five-wk-old male Sprague–Dawley (SD) rats (*n* = 30) were obtained from Chubu Kagakushizai (Nagoya, Japan) and were maintained on standard chow and water ad libitum and housed in individual cages (260 × 382 × 200 mm, 3 rats in each cage) under controlled temperatures (24 ± 1.0°C) with a 12 h light/dark cycle. The rats were divided into 3 groups: normal, periodontitis, and periodontitis with liraglutide treatment groups. The primary outcomes were inflammatory cell infiltration into the gingiva, gene expressions of the inflammatory cytokines in the gingiva, and alveolar bone loss. The sample size was determined using G∗Power 3.1 [16]. This study was approved by the Institutional Animal Care and Use Committees of Aichi Gakuin University (AGUD429), and all animal experiments were conducted following the national guidelines and the relevant national laws on the protection of animals.

### 2.2. Induction of Experimental Periodontitis by Ligation

**Figure 1:**
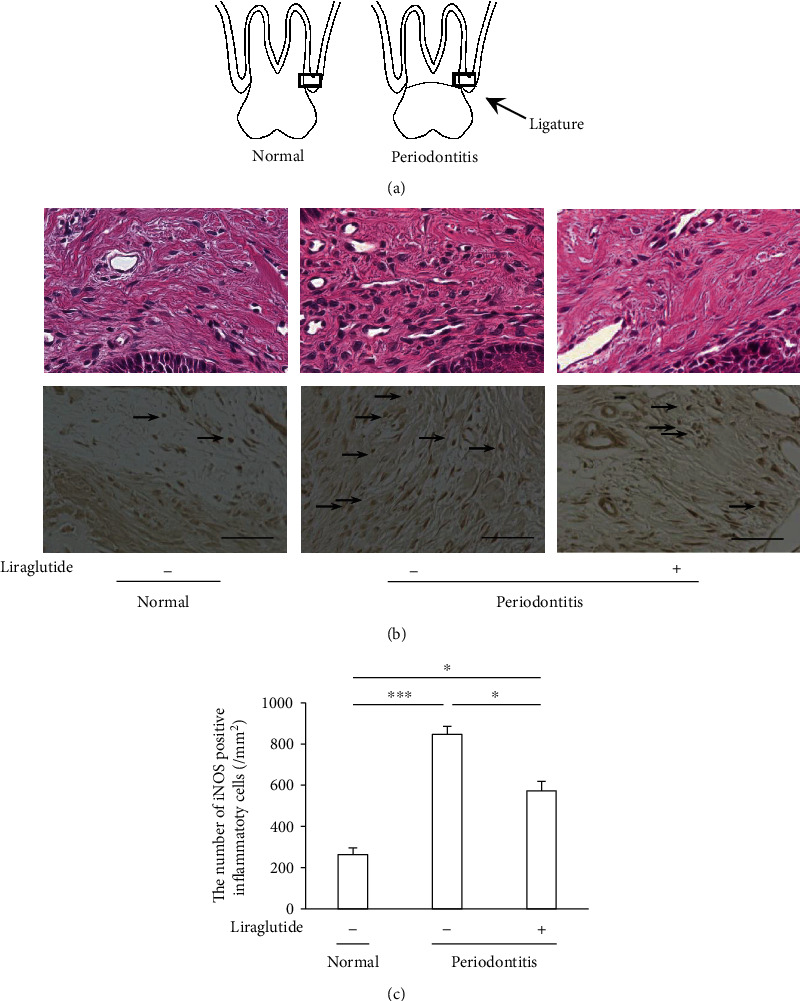
Histological evaluation of the gingiva. Liraglutide decreased the inflammatory cell invasion in periodontitis. (a) Schematic images of periodontal tissue with (periodontitis) and without (normal) ligation. The squares represent the parts shown in the below figures. (b) Upper panels: H&E staining of the periodontal tissues around the tooth 14 days after ligation. Lower panels: detection of iNOS-positive inflammatory cells in the gingiva. The black arrows mark iNOS-positive inflammatory cells. Scalebar = 50 *μ*m. (c) Quantification of iNOS-positive inflammatory cells in the gingiva. The results are expressed as the mean ± SEM (*n* = 6). ^∗^*P* < 0.05 and ^∗∗∗^*P* < 0.001.

The rats were deeply anaesthetized with mixed anesthetic agents consisting of medetomidine hydrochloride, midazolam, and butorphanol tartrate. Surgical nylon thread (3-0 Surgilon; Tyco Healthcare, Princeton, NJ, USA) was ligated around the cervical portion of the maxillary second molar to induce experimental periodontitis as previously described (Figure 1(a)) [17]. Fourteen days after the induction of periodontitis, the rats were sacrificed with CO_2_ and periodontitis was evaluated as described below.

### 2.3. Administration of Liraglutide

Half of the rats with ligature for the induction of periodontitis were sustainably administered liraglutide (Victoza; Novo Nordisk, Copenhagen, Denmark) (0.03 mg/kg/day) via the osmotic pump (Durect Corporation, Cupertino, CA, USA) (pumping rate 0.5 *μ*L/h (±0.1 *μ*l/h)) for 14 days from the day of the ligature. The pump was filled with liraglutide and implanted subcutaneously in the back of the rats. The pump delivered the solution continuously for 14 days without the need for external connections. The osmotic pump filled with saline was inserted in each control rat.

### 2.4. Tissue Collection

For mRNA analyses, gingival mucosal tissues were excised from the buccal side of the second molar on both sides. The samples were immediately submerged in RNAlater RNA Stabilization Reagent (Qiagen, Valencia, CA, USA) and stored at -­80°C until use. For immunohistological staining and microcomputed tomography (micro-CT) analyses, the maxillary bones with gingival tissues attached on both sides were fixed in 10% formalin.

### 2.5. Gene Expression Analyses

Total RNA was extracted using a RNeasy Mini Kit (Qiagen). Complementary DNA was synthesized from RNA using ReverTra Ace (Toyobo, Osaka, Japan). The primers were purchased from TaqMan Gene Expression Assays (Applied Biosystems, Foster City, CA, USA). Real-time quantitative polymerase chain reaction (PCR) was performed using a LightCycler 480 system (Roche Diagnostics, Basel, Switzerland).

### 2.6. Pathological and Immunohistological Evaluation of Periodontal Tissues

The fixed periodontal tissues including alveolar bones were decalcified in 10% ethylenediaminetetraacetic acid (EDTA) for 5 weeks and embedded in paraffin and cut by 5 *μ*m. The sections were stained with hematoxylin and eosin. For the analyses of inflammatory cell infiltration, the sections were incubated with anti-inducible nitric oxide synthase (iNOS) antibody (Bioss, Boston, USA). Although iNOS is expressed mainly in inflammatory cells, other type of cells such as vascular smooth muscle cells express iNOS in some cases. Therefore, we calculated the number of iNOS-positive rounded cells as iNOS-positive inflammatory cells under light microscopy (×400) by one skilled investigator.

To observe the osteoclasts and osteoblasts, the sections were stained with tartrate-resistant acid phosphatase (TRAP) and alkaline phosphatase (ALP) (Sept. Sapie Co., Ltd., Tokyo, Japan). Osteoclasts were defined as multinucleated, TRAP-positive cells in contact with the surface of the alveolar bone [18]. The activities of osteoblasts were evaluated by the intensity of ALP using ImageJ. The number of TRAP-positive cells and the intensity of ALP per the length of alveolar bone surface were calculated.

### 2.7. Analysis of Alveolar Bone Resorption

For the evaluation of alveolar bone loss, alveolar bone was scanned by micro-CT (Cosmo Scan GX; Rigaku Corporation, Tokyo, Japan) 2 weeks after the ligature. Three-dimensional images were generated using TRI/3D BON (Ratoc, Tokyo, Japan). Bone resorption was blindly calculated as the distance from the mesial buccal cementoenamel junction to the alveolar bone crest by two skilled investigators.

### 2.8. Statistical Analyses

The data are expressed as the means ± standarderror of the mean (SEM). The data sets were assessed by analysis of variance (one-way ANOVA) followed by the Bonferroni correction for multiple comparisons. The differences were considered significant when *P* < 0.05.

## 3. Results

### 3.1. Animal Characteristics

At baseline and two weeks after the induction of periodontitis with or without administration of liraglutide, body weights and blood glucose concentrations were not significantly changed between the three groups, indicating that periodontitis and GLP-1 did not affect these systemic parameters (Table 1). The number of animals in each group was different due to death in the experimental term after the ligation of second molar.

### 3.2. Liraglutide Ameliorated Periodontitis-Induced Infiltration of Inflammatory Cells into the Gingiva

Two weeks after the ligation, periodontal tissues were histologically evaluated. Inflammatory cells were infiltrated into the gingiva by periodontitis (Figure 1(b)). Liraglutide ameliorated the periodontitis-induced inflammatory cell infiltration in the gingiva.

Because inflammatory cells express iNOS, we evaluated iNOS-positive cells by immunohistological staining (Figure 1(b)). iNOS-positive cells were significantly increased by periodontitis, which was decreased by liraglutide. Quantitative analysis demonstrated that the number of iNOS-positive cells in the gingiva was increased by 3.3-fold compared with that in the normal gingiva (*P* < 0.001), which was significantly decreased by liraglutide by 40% (*P* < 0.05) (Figure 1(c)).

### 3.3. Liraglutide Decreased Periodontitis-Induced Inflammatory Gene Expressions in the Gingiva

**Figure 2:**
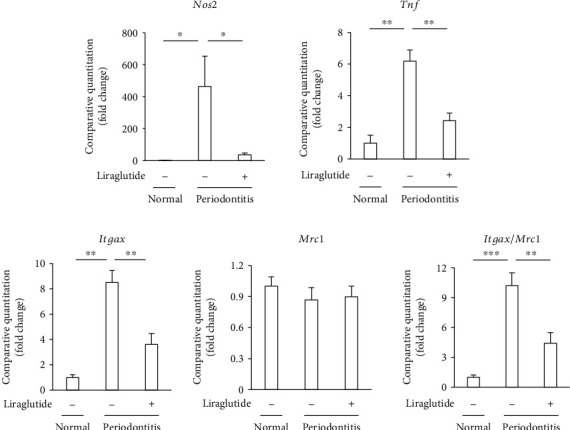
Liraglutide attenuated the gene expressions of inflammatory cytokines induced by periodontitis. Gene expressions of iNOS (*Nos2*), TNF-*α* (*Tnf*), CD11c (*Itgax*), and CD206 (*Mrc1*) were determined by quantitative real-time PCR. The results are expressed as the mean ± SEM (*n* = 5). ^∗^*P* < 0.05, ^∗∗^*P* < 0.01, and ^∗∗∗^*P* < 0.001.

Periodontitis significantly increased the gene expressions of iNOS (*Nos2*) and tumor necrosis factor-*α* (TNF-*α*) (*Tnf*) by 450-fold and 6.0-fold, respectively, compared to normal rats (Figure 2). Liraglutide significantly decreased periodontitis-induced iNOS and TNF-*α* gene expressions by 92% and 72%, respectively, in the gingiva.

Because TNF-*α* and iNOS are M1 macrophage-related inflammatory genes, we also investigated the polarization of macrophages in the gingiva by evaluating the surface markers of M1 and M2 macrophages. Periodontitis significantly increased the gene expression of the M1 macrophage-related surface marker, CD11c (*Itgax*), which was significantly decreased by liraglutide. On the other hand, the expression of the M2 macrophage surface marker, CD206 (*Mrc1*), was unchanged by periodontitis or liraglutide-treated periodontitis. These results suggest that liraglutide ameliorates periodontitis by reducing M1 macrophage infiltration into the gingiva.

### 3.4. Liraglutide Reduced Alveolar Bone Resorption by Periodontitis

**Figure 3:**
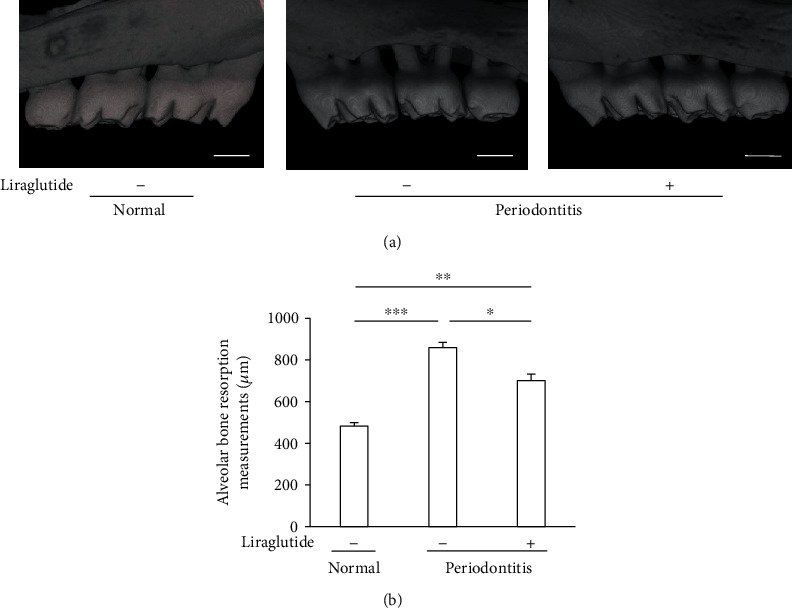
Liraglutide reduced bone resorption in rats with periodontitis. (a) Generated three-dimensional images of alveolar bone. Scalebar = 1 mm. (b) Bone resorption was calculated as the distance from the buccal cementoenamel junction to the alveolar bone crest. The results are expressed as the mean ± SEM (*n* = 8-10). ^∗^*P* < 0.05, ^∗∗^*P* < 0.01, and ^∗∗∗^*P* < 0.001.

The micro-CT images of the buccal side of the second molars in normal, periodontitis, and periodontitis + liraglutide rats are shown in Figure 3(a). Bone resorption was significantly augmented in the periodontitis rats compared with normal rats by 1.8-fold (*P* < 0.001) (Figure 3(b)). Liraglutide reduced alveolar bone resorption in the periodontitis rats by 42% (*P* < 0.05).

We evaluated the correlation between the number of iNOS-positive inflammatory cells in the gingiva and alveolar bone resorption (Supplemental Figure 1). There was a positive linear correlation between iNOS-positive inflammatory cells in the gingiva and alveolar bone resorption (*r*^2^ = 0.73), suggesting that alveolar bone loss was induced by the inflammation of gingiva.

### 3.5. Liraglutide Decreased Periodontitis-Induced Osteoclasts on the Surface of Alveolar Bone

**Figure 4:**
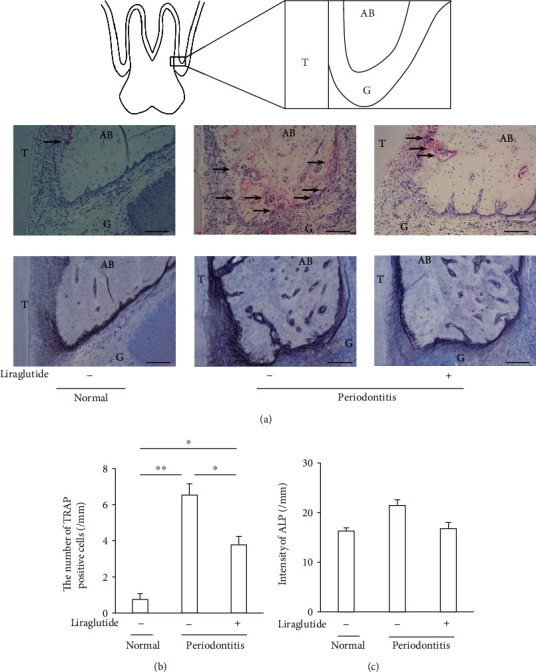
Evaluation of osteoclast formation and osteoblast activity on the surface of alveolar bone. Histologic sections were obtained 14 days after ligation and administration of liraglutide. (a) Upper panels: pattern diagrams of anatomical aspects of alveolar tissues. T: tooth; G: gingiva; AB: alveolar bone. Middle panels: TRAP-positive osteoclast localization in the second molar of the maxillary frontal sections; the black arrows mark the TRAP-positive cells. Scalebar = 100 *μ*m. Lower panels: ALP staining of the same part as upper panels. Scalebar = 100 *μ*m. (b) Quantification of TRAP-positive cells (osteoclast) (nuclei of ≧3). (c) Quantification of ALP (activity of osteoblasts) by ImageJ. The results are expressed as the mean ± SEM (*n* = 3). ^∗^*P* < 0.05 and ^∗∗^*P* < 0.01.

Osteoclasts were identified by TRAP staining. As shown in Figures 4(a) and 4(b), the induction of periodontitis exhibited a marked increase in osteoclasts on the surface of alveolar bone by 8.7-fold compared to normal rats (*P* < 0.01). The number of osteoclasts on the alveolar bone was significantly decreased in liraglutide-treated periodontitis rats by 47.5% (*P* < 0.05). The viability of osteoblasts was assessed by ALP activity (Figure 4(a)). Periodontitis tended to increase osteoblasts, which was cancelled by the liraglutide treatment; however, these were not significant (Figure 4(c)).

## 4. Discussion

We demonstrated the effects of a GLP-1 receptor agonist liraglutide on the development of periodontitis. Ligature-induced periodontitis was identified by the infiltration of inflammatory cells and increased inflammatory gene expressions in the gingiva and alveolar bone loss in rats, all of which were reduced by liraglutide. We further revealed that liraglutide decreased periodontitis-related inflammatory M1 macrophages in the gingiva and osteoclasts on the surface of alveolar bone.

We previously demonstrated that the induction of periodontitis in GIP receptor knockout mice was more severe than that in wild-type mice and was accompanied with aggregated macrophages in the gingiva [9], which demonstrates the beneficial effects of GIP on periodontitis. Although GIP receptor agonists are still under clinical trial, GLP-1 receptor agonists have been widely used for the therapy of patients with type 2 diabetes as well as patients with obesity. Here, we demonstrated that liraglutide ameliorated the development of periodontitis by ligature independent of glucose-lowering effect and antiobesity effect, which suggest additive effects of the treatment of periodontitis in patients taking liraglutide. Furthermore, the beneficial effects of GLP-1 on periodontitis indicate the possibility of new therapeutic strategy for periodontitis.

The anti-inflammatory effects of GLP-1 receptor agonists were demonstrated in several cells and disease models. Liraglutide inhibited lipopolysaccharide-induced TNF-*α* expressions in vascular endothelial cells and monocytes [19]. A GLP-1 receptor agonist exendin-4 significantly diminished the brain injury-induced overexpression of TNF*α* and interleukin-1*β* (IL-1*β*) [20]. Another GLP-1 receptor agonist exenatide inhibited TNF-*α* and monocyte chemotactic protein-1 (MCP-1) levels and F4/80 expression in the testes of mice with high-fat diet-induced obesity [21]. We demonstrated that two-week administration of liraglutide ameliorated ligature-induced inflammatory cell infiltration and gene expression in the gingiva. These anti-inflammatory effects on periodontitis are consistent with other GLP-1 agonists. For example, exenatide decreased the periodontitis-induced inflammatory gene expression of IL-1*β*, iNOS, and *matrix metalloproteinase-9* (MMP-9) in the gingiva [22].

Macrophages are polarized to the M1 state when stimulated by TNF-*α*, interferon-*γ* (IFN-*γ*), or other bacterial products and to the M2 state when stimulated by IL-4, IL-13, and IL-10 [23]. Macrophage polarization in periodontitis is controversial. Most investigations of both rodent models and human periodontitis demonstrated that M1 macrophage is predominant and the M1/M2 ratio is increased in periodontitis [24–26]. On the other hand, there is a report that the polarization of macrophages in the gingiva was similar between periodontitis subjects and healthy subjects [27]. Here, we demonstrated that M1 macrophages were significantly increased in periodontitis, although M2 macrophages did not show significant change between normal tissue and periodontitis tissue. M1/M2 ratio was significantly increased in periodontitis compared with normal tissue. Liraglutide reduced M1 macrophage, which resulted in the reduction of M1/M2 ratio, which was partially consistent with previous studies. It was reported that GLP-1 inhibited M1 activation and induced M2 activation via the cyclic adenosine monophosphate/protein kinase A pathway in RAW 264.7 macrophages [13]. Another GLP-1 analogue lixisenatide showed antiatherosclerotic effects with the reduction of M1 macrophages and increase of M2 macrophages in atheroma [28].

From the current results, it is considered that osteoclastogenesis is increased in the periodontitis group. Since there is a literature that ALP expression is increased around the lesion where inflammatory bone resorption occurs, it might be possible to consider that bone metabolism is accelerated in the bone environment around the inflammatory lesion [29]. The expression of GLP-1 receptors has been reported in murine osteoblasts and osteoclasts as well as human osteoblastic cell lines [30–32]. We demonstrated that periodontitis-induced osteoclastogenesis was downregulated by liraglutide. On the contrary, we could not observe the increase of osteoblast activity by liraglutide, indicating that the impact of liraglutide on alveolar bone was mainly due to the decrease of osteoclast formation. Because TNF-*α* induces osteoclast formation directly and indirectly, the anti-inflammatory effects of GLP-1 may play a crucial role in the decrease of osteoclast formation [33, 34]. A positive correlation between gingival inflammation and alveolar bone resorption in our study suggests the importance of inflammatory regulation in periodontitis. On the other hand, exenatide did not reduce periodontitis-related alveolar bone loss, but a longer period of administration was reported to ameliorate bone cortical microarchitecture and bone compositional parameters in high-fat diet-fed diabetic mice [22]. Additional studies are required to clarify the drug effects among the GLP-1 receptor agonists on periodontitis-related alveolar bone loss.

As described previously, the morbidity of periodontitis is high and the level of periodontitis is severer than nondiabetic subjects. An epidemiological study demonstrated that good glycemic control (HbA1c < 7.0%) diminished the aggravation of periodontitis compared with that of poor glycemic control in both type 1 and type 2 diabetes although the levels of attachment loss, one of the parameters of periodontitis, were still higher than nondiabetic subjects [35]. Obesity is also correlated with periodontitis [36]. Since GLP-1 receptor agonists are used for the treatment of type 2 diabetes worldwide and for patients with obesity in several countries, our results make us expect that GLP-1 receptor agonists have an additive effect to treat periodontitis in addition to its own glucose-lowering and body weight-lowering effects in patients with type 2 diabetes or obesity.

In conclusion, we revealed that liraglutide ameliorates the development of periodontitis, which was demonstrated by the reduction of periodontitis-induced inflammation accompanied with the decrease of M1 macrophages in the gingiva and the reduction of alveolar bone loss with decreased osteoclast formation in this study. Our results showed the direct effects of the GLP-1 receptor as a treatment for periodontitis, separately from the glucose-lowering and antiobesity effects.

## Figures and Tables

**Table 1:** Body weights and blood glucose levels in normal, periodontitis, and liraglutide-treated periodontitis rats.

Variable	Normal (*n* = 8)	Periodontitis (*n* = 8)	*P* value	Periodontitis + liraglutide (*n* = 7)	*P* value
Pretreatment					
Body weight (g)	191.0 ± 3.6	188.0 ± 7.7	>0.999	188.0 ± 5.9	>0.999

Posttreatment					
Body weight (g)	313.1 ± 11.6	309.3 ± 9.7	>0.999	294.1 ± 8.3	0.705
Blood glucose (mmol/L)	5.9 ± 1.3	5.3 ± 1.0	>0.999	5.8 ± 1.4	>0.999

Pretreatment: the day of the ligature. Posttreatment: two weeks after the ligature with or without liraglutide. Data are means ± standarderror. *P* value versus normal rats.

## Data Availability

No data were used to support this study.

## Supplementary Materials

Supplementary materials Supplemental Figure 1: the correlation between the number of iNOS-positive inflammatory cells in the gingiva and alveolar bone resorption. There was a positive linear correlation between iNOS-positive inflammatory cells in the gingiva and alveolar bone resorption (*r*^2^ = 0.73).

## References

[1] Papapanou P. N., Sanz M., Buduneli N. (2018). Periodontitis: Consensus report of workgroup 2 of the 2017 World Workshop on the Classification of Periodontal and Peri-Implant Diseases and Conditions. *Journal of Periodontology*.

[2] Löe H. (1993). Periodontal disease. The sixth complication of diabetes mellitus. *Diabetes Care*.

[3] Khader Y. S., Dauod A. S., El-Qaderi S. S., Alkafajei A., Batayha W. Q. (2006). Periodontal status of diabetics compared with nondiabetics: a meta-analysis. *Journal of Diabetes and its Complications*.

[4] Borgnakke W. S., Yl€ostalo P. V., Taylor G. W., Genco R. J. (2013). Effect of periodontal disease on diabetes: systematic review of epidemiologic observational evidence. *Journal of Periodontology*.

[5] Graziani F., Gennai S., Solini A., Petrini M. (2018). A systematic review and meta-analysis of epidemiologic observational evidence on the effect of periodontitis on diabetes an update of the EFP-AAP review. *Journal of Clinical Periodontology*.

[6] Teeuw W. J., Gerdes V. E. A., Loos B. G. (2010). Effect of periodontal treatment on glycemic control of diabetic patients: a systematic review and meta-analysis. *Diabetes Care*.

[7] Simpson T. C., Weldon J. C., Worthington H. V. (2015). Treatment of periodontal disease for glycaemic control in people with diabetes mellitus. *Cochrane Database of Systematic Reviews*.

[8] Saremi A., Nelson R. G., Tulloch-Reid M. (2004). Periodontal Disease and Mortality in Type 2 Diabetes. *Diabetes Care*.

[9] Suzuki Y., Nakamura N., Miyabe M. (2016). Anti-inflammatory role of glucose-dependent insulinotropic polypeptide in periodontitis. *Journal of Diabetes Investigation*.

[10] Seino Y., Yabe D. (2013). Glucose-dependent insulinotropic polypeptide and glucagon-like peptide-1: incretin actions beyond the pancreas. *Journal of Diabetes Investigation*.

[11] Davies M. J., D’Alessio D. A., Fradkin J. (2018). Management of hyperglycaemia in type 2 diabetes, 2018. A consensus report by the American Diabetes Association (ADA) and the European Association for the Study of Diabetes (EASD). *Diabetologia*.

[12] Lee Y.-S., Jun H.-S. (2016). Anti-inflammatory effects of glp-1-based therapies beyond glucose control. *Mediators of Inflammation*.

[13] Wan S., Sun H. (2019). Glucagon-like peptide-1 modulates RAW264.7 macrophage polarization by interfering with the JNK/STAT3 signaling pathway. *Experimental and Therapeutic Medicine*.

[14] Sun H. X., Lu N., Luo X., Zhao L., Liu J. M. (2015). Liraglutide, the glucagon-like peptide-1 receptor agonist, has anabolic bone effects in diabetic Goto-Kakizaki rats. *Journal of Diabetes*.

[15] Yamada C., Yamada Y., Tsukiyama K. (2008). The murine glucagon-like peptide-1 receptor is essential for control of bone resorption. *Endocrinology*.

[16] Faul F., Erdfelder E., Lang A. G., Buchner A. (2007). GPower 3: a flexible statistical power analysis program for the social, behavioral, and biomedical sciences. *Behavior Research Methods*.

[17] Adachi K., Miyajima S. I., Nakamura N. (2017). Role of poly (ADP-ribose) polymerase activation in the pathogenesis of periodontitis in diabetes. *Journal of Clinical Periodontology*.

[18] Iguchi S., Suzuki D., Kawano E. (2017). Effect of local bone marrow stromal cell administration on ligature-induced periodontitis in mice. *Journal of Oral Science*.

[19] Krasner N. M., Ido Y., Ruderman N. B., Cacicedo J. M. (2014). Glucagon-like peptide-1 (GLP-1) analog liraglutide inhibits endothelial cell inflammation through a calcium and AMPK dependent mechanism. *PLoS One*.

[20] Zhang J., Yi T., Cheng S., Zhang S. (2020). Glucagon-like peptide-1 receptor agonist Exendin-4 improves neurological outcomes by attenuating TBI- induced inflammatory responses and MAPK activation in rats. *International Immunopharmacology*.

[21] Insuela D. B. R., Carvalho V. F. (2017). Glucagon and glucagon-like peptide-1 as novel anti-inflammatory and immunomodulatory compounds. *European Journal of Pharmacology*.

[22] Moraes R. M., Lima G. M. G., Oliveira F. E. (2015). Exenatide and sitagliptin decrease interleukin 1*β*, matrix metalloproteinase 9, and nitric oxide synthase 2 gene expression but does not reduce alveolar bone loss in rats with periodontitis. *Journal of Periodontology*.

[23] Martinez F. O. (2008). Macrophage activation and polarization. *Frontier in Bioscience*.

[24] Yu T., Zhao L., Huang X. (2016). Enhanced activity of the macrophage M1/M2 phenotypes and phenotypic switch to M1 in periodontal infection. *Journal of Periodontology*.

[25] Zhou L.‐. N., Bi C.‐. S., Gao L.‐. N., An Y., Chen F., Chen F.‐. M. (2018). Macrophage polarization in human gingival tissue in response to periodontal disease. *Oral Diseases*.

[26] Yang J., Zhu Y., Duan D. (2018). Enhanced activity of macrophage M1/M2 phenotypes in periodontitis. *Archieves of Oral Biology*.

[27] Garaicoa-Pazmino C., Fretwurst T., Squarize C. H. (2019). Characterization of macrophage polarization in periodontal disease. *Journal of Clinical Periodontology*.

[28] Vinué Á., Navarro J., Herrero-Cervera A. (2017). The GLP-1 analogue lixisenatide decreases atherosclerosis in insulin-resistant mice by modulating macrophage phenotype. *Diabetologia*.

[29] Anan H., Akamine A., Hara Y., Maeda K., Hashiguchi I., Aono M. (1991). A histochemical study of bone remodeling during experimental apical periodontitis in rats. *Journal of Endodontics*.

[30] Aoyama E., Watari I., Podyma-Inoue K. A., Yanagishita M., Ono T. (2014). Expression of glucagon-like peptide-1 receptor and glucose-dependent insulinotropic polypeptide receptor is regulated by the glucose concentration in mouse osteoblastic MC3T3-E1 cells. *International Journal of Molecular Medicine*.

[31] Pereira M., Jeyabalan J., Jørgensen C. S. (2015). Chronic administration of Glucagon-like peptide-1 receptor agonists improves trabecular bone mass and architecture in ovariectomised mice. *Bone*.

[32] Pacheco-Pantoja E. L., Ranganath L. R., Gallagher J. A., Wilson P. J. M., Fraser W. D. (2011). Receptors and effects of gut hormones in three osteoblastic cell lines. *BMC Physiology*.

[33] Abu-Amer Y. (2000). Tumor necrosis factor receptors types 1 and 2 differentially regulate osteoclastogenesis. *Journal of Biological Chemistry*.

[34] Abu-Amer Y., Ross F. P., Edwards J., Teitelbaum S. L. (1997). Lipopolysaccharide-stimulated osteoclastogenesis is mediated by tumor necrosis factor via its P55 receptor. *The Journal of Clinical Investigation*.

[35] Demmer R. T., Holtfreter B., Desvarieux M. (2012). The influence of type 1 and type 2 diabetes on periodontal disease progression: prospective results from the Study of Health in Pomerania (SHIP). *Diabetes Care*.

[36] Wood N., Johnson R. B., Streckfus C. F. (2003). Comparison of body composition and periodontal disease using nutritional assessment techniques: Third National Health and Nutrition Examination Survey (NHANES III). *Journal of Clinical Periodontology*.

