# Supplementary figures and images for: Glucagon-Like Peptide-1 Receptor Agonist Liraglutide Ameliorates the Development of Periodontitis
Source: J Diabetes Res. 2020 Nov 19;2020:8843310. doi: 10.1155/2020/8843310 (PMC7695495; doi:10.1155/2020/8843310)

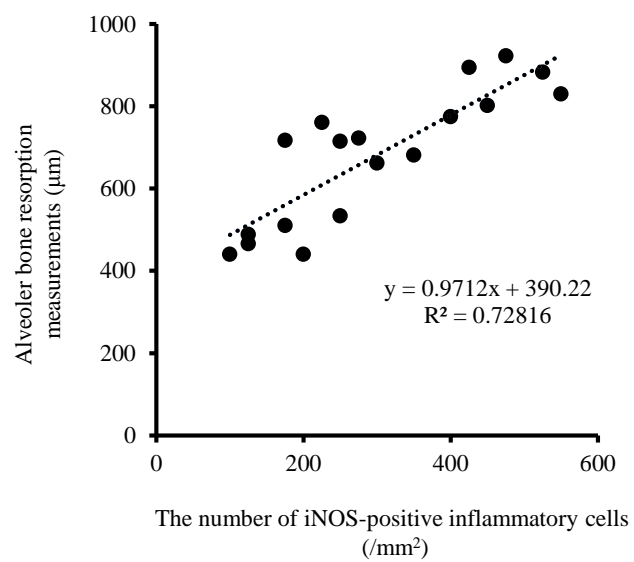

SUPPLEMENTAL FIGURE 1.

Supplement: Supplementary materials — Supplemental Figure 1: the correlation between the number of iNOS-positive inflammatory cells in the gingiva and alveolar bone resorption. There was a positive linear correlation between iNOS-positive inflammatory cells in the gingiva and alveolar bone resorption (r2 = 0.73). [file 8843310.f1.pdf]
